# Freshwater-Derived *Streptomyces*: Prospective Polyvinyl Chloride (PVC) Biodegraders

**DOI:** 10.1155/2022/6420003

**Published:** 2022-11-14

**Authors:** Maria Fernanda Rodríguez-Fonseca, Sonia Ruiz-Balaguera, Manuel Fernando Valero, Jeysson Sánchez-Suárez, Ericsson Coy-Barrera, Luis Eduardo Díaz

**Affiliations:** ^1^Process Design and Management, School of Engineering, Universidad de La Sabana, Chía 250001, Colombia; ^2^Bioprospecting Research Group, School of Engineering, Universidad de La Sabana, Chía 250001, Colombia; ^3^Conservation, Bioprospecting, and Sustainable Development Group, Environmental Engineering Program, Universidad Nacional Abierta y a Distancia (UNAD), Bogotá 110911, Colombia; ^4^Energy, Materials and Environment Group, School of Engineering, Universidad de La Sabana, Chía 250001, Colombia; ^5^Environmental Engineering Program, School of Exact Sciences and Engineering, Universidad Sergio Arboleda, Bogotá 111071, Colombia; ^6^Bioorganic Chemistry Laboratory, Universidad Militar Nueva Granada, Cajicá 250247, Colombia

## Abstract

Polyvinyl chloride (PVC) is widely used in industrial applications, such as construction and clothing, owing to its chemical, physical, and environmental resistance. Owing to the previous characteristics, PVC is the third most consumed plastic worldwide and, consequently, an increasing waste accumulation-related problem. The current study evaluated an in-house collection of 61 Actinobacteria strains for PVC resin biodegradation. Weight loss percentage was measured after the completion of incubation. Thermo-gravimetric analysis was subsequently performed using the PVC incubated with the three strains exhibiting the highest weight loss. GC-MS and ionic exchange chromatography analyses were also performed using the culture media supernatant of these three strains. After incubation, 14 strains had a PVC weight loss percentage higher than 50% in ISP-2 broth. These 14 strains were identified as Streptomyces strains. Strains 208, 250, and 290 showed the highest weight loss percentages (57.6–61.5% range). The thermal stability of PVC after bacterial exposure using these three strains was evaluated, and a modification of the representative degradation stages of nonincubated PVC was observed. Additionally, GC-MS analysis revealed the presence of aromatic compounds in the inoculated culture media, and ionic exchange chromatography showed chloride release in the supernatant. A mathematical relation between culture conditions and PVC weight loss was also found for strains 208 and 290, showing an accuracy up to 97.99%. These results highlight the potential of the freshwater-derived Streptomyces strains as candidates for the PVC biodegradation strategy and constitute the first approach to a waste management control scale-up process.

## 1. Introduction

Commodity plastics are one of the most commonly used materials, and their production is rapidly increasing and is expected to reach 500 million tons by the end of 2020 [1]. This enhanced demand has led to a significant increase in waste production and accumulation [1, 2]. Polyvinyl chloride (PVC) is the third most consumed plastic worldwide [3]. In 2017, its consumption in the sole packaging industry reached 5 million tons in Europe [4] and tended to increase. PVC is widely known for its chemical/physical resistance, which is one of the main reasons for its importance in diverse industries [3]. Owing to this resistance, PVC waste is one of the hardest materials to degrade, as these polymers can last up to 200 years in the environment without any treatment [5]. Accordingly, PVC waste management is an important issue regarding pollution [1].

According to environmental organizations, less than 12% of PVC waste is recycled in Colombia [6], primarily due to incorrect waste disposal [5, 6]. As recycling PVC waste is limited, different degradation methods have been used for PVC waste management, such as thermal and burial degradation [7, 8]. These methods are highly used in landfills; however, they are accompanied by problems owing to material resistance and composition [7]. With thermal degradation (i.e., pyrolysis), up to 39.5% of the total plastic waste in landfills can be degraded [7]. For this approach, high temperatures must be used (up to 525°C) [7], and the most common by-products include aromatic compounds, hydrogen chloride, carbon dioxide, and halogenated compounds [9]. With burial degradation, waste accumulation tends to be a long-term problem. Similarly, these wastes produce leachate that can pollute underground water with highly toxic compounds, such as dioxins, that tend to bioaccumulate [10, 11]. These by-products are harmful and toxic to human health and are environmental pollutants [10].

There is an increasing need to identify a complementary method with less or no adverse effects on the environment and human health [10]. Biodegradation has been studied as an alternative method for plastic waste management due to its low pollution levels and harmful by-product formation [12]. The main concern with biodegradation is the inability of plastics to be 100% degraded. Furthermore, pretreatment is often needed to carry out the degradation [13]; studies have revealed high percentages (*ca.* 68%) of biodegradation with different commonly-used plastics, such as high-density polyethylene (HDPE) and polyethylene terephthalate (PET) [13, 14]. With biopolymers, some microorganisms have shown a positive biodegradation performance [15, 16], such as polycaprolactone (PCL) and polyhydroxyalkanoate (PHA), reaching 13% and 17% of weight loss after bacterial exposure (4 to 8 weeks) [17]. In some cases, the enzyme responsible for this degradation has been isolated and characterized, as revealed in the US patent US8728776B2, where a bacterial strain with polylactic acid degradation was isolated. The enzyme responsible for this degradation was characterized as a depolymerase [18]. To date, the PVC biodegradation process has not been studied to the same extent as the biodegradation of other commodity plastics. Some bacteria and fungi, such as *Pseudomonas citronellolis*, *Bacillus flexus,* and *Chaetomium globosum*, have been tested as part of biodegradation procedures using PVC films, with biodegradation rates of 13%, 18%, and 9%, respectively, measured as the weight loss after ca. 30-day microbial exposure [19, 20].

As biodegradation processes are often associated with enzyme production, bacterial strains with diverse biological activities are targeted as potential solutions for biologically controlling xenobiotic compounds [21]. Species such as *Streptomyces* spp. are highly abundant in the environment and have been studied for decades owing to their wide secondary metabolism, which includes a rich range of enzymes; their use at the industrial level, such as antibiotic production for the pharmaceutical industry [22], and their remarkable adaptive behavior according to the environment in [23]. *Streptomyces* has been studied for the biodegradation of many substrates, such as pesticides, heavy metals, biopolymers, and commodity plastics [13, 24]. Regarding commodity plastics, there are reports on HDPE and PET biodegradation, both using powdered wastes as pretreatment. Some positive results have also been obtained for HDPE and PET degradation, with 18% and 68.8% weight loss after bacterial exposure [13, 14]. In these cases, an incubation period of 18 days was used. In these reports, powdered plastic wastes with different particle sizes were used, with better results obtained with lower particle sizes (e.g., 212 *μ*m). Furthermore, the target plastic was the main carbon source in the culture media [13, 14].

As biodegradation seems to be an alternative and complementary method for PVC waste control and management [25], there is a constant need to find the microorganisms and conditions that allow this process to take place [25]. One of the main approaches to determining the culture conditions that allow a better bacterial response (e.g., weight loss and enzyme production) is to mathematically relating or modeling the bacterial behavior under controlled conditions. However, the understanding of the plastics biodegradation process is a recent study, and optimization of culture conditions for PVC biodegradation by Actinobacteria or *Streptomyces* strains has not been done yet. To our knowledge, different kinetic models have been used to describe *Streptomyces* behavior on plastics biodegradation [13], but no research on culture conditions optimization or mathematical relation with biodegradation capacity has been done so far.

Based on a previously conducted systematic review, no studies evaluating PVC biodegradation by *Streptomyces* have been conducted [26] despite the positive results of *Streptomyces* for commodity plastics and biopolymer biodegradation [16]. Thus, there is an unexplored potential of these bacteria for PVC biodegradation. In this study, we evaluated the biodegradation capacity of freshwater-derived *Streptomyces* under standard culture conditions. Briefly, several *Streptomyces* strains (61) from the CIBUS collection at the Bioprospecting Research Group (GIBP) were evaluated for PVC biodegradation. These strains were selected to determine their potential for biodegradation to fully characterize previously examined strains, from Arauca and Guaviare riverbanks, by GIBP with diverse biological activities [23, 27]. Also, the effects of culture conditions on two selected strains were evaluated and determined using Plackett–Burman design, and a suitable mathematical relation by Box–Behnken design was also determined for PVC weight loss percentage.

## 2. Materials and Methods

### 2.1. Chemicals

Bacteriological agar (Oxoid, Hampshire, UK), malt extract, yeast extract, glucose, ethanol, ammonium sulfate, sodium nitrate, monopotassium phosphate, disodium hydrogen phosphate (ITW Reagents, Hannover, Germany), ethyl acetate, calcium nitrate, and magnesium sulfate (Merck and Co, Darmstadt, Germany) were employed in this study. PVC resin with no plasticizers (particle size up to 150 *μ*m) was provided by Grupo PVC Compuestos y Resinas S.A.S (Bogotá, Colombia).

### 2.2. Biological Material

An in-house collection comprising sixty-one (61) Actinobacteria-like strains previously isolated from Arauca (7°04′21.5″N 70°49′38.7″W) and Guaviare (18N 7°57.697E 2°86.555N) riverbanks with similar morphology to the species *Streptomyces* was selected and its capability of PVC biodegradation was determined.

### 2.3. Culture Media and Culture Conditions

Three culture media were prepared as follows: (1) ISP-2 agar, (2) modified ISP2- broth, and (3) modified mineral salt medium (MSM). The components of each culture medium are listed in Table 1.

All culture media were prepared for further analysis using deionized (DI) water. Strain activation was carried out via massive sowing on ISP-2 agar (media used for strain isolation/preservation) from cryopreserved inoculum, with ten days of incubation at 30°C. The growing strains were considered for PVC biodegradation screening. For each strain, a biomass stock solution was prepared in ISP-2 broth at 30°C and 200 rpm. The modified ISP-2 broth was used as the adaptation/screening stage. Modifications to MSM were carried out to prevent chloride interference in subsequent analyses and the influence of other carbon sources on the bacterial strains. Tween 80 was added to prevent bacterial agglomeration. All culture media were adjusted to a pH of 7.01.

### 2.4. Selection of Positive Strains for PVC Biodegradation

Strains were cultivated on ISP-2 modified medium using a plug from an agar plate (*ca*. 10 mm in diameter) to determine PVC biodegradation performance under standard culture conditions. The following standard conditions were determined based on previous research [23, 27]: 1 mL (*ca.* 0.2 mg) of biomass was taken from the stock and inoculated on modified ISP-2 broth at 30°C, 200 rpm, pH 7.0, with a total volume of 10 mL, and a PVC particle size of 150 *μ*m for 18 days. After incubation, the PVC weight loss was measured, and strains that achieved ≥50% were selected for further analysis. After the PVC degrading strains were selected, MSM was used to assess PVC biodegradation on a medium with PVC as the sole carbon source under standard aerobic conditions described before; the MSM volume was 20 mL. Test tubes with PVC were autoclaved at 120°C for 15 min, cooled to room temperature, and then inoculated with 500 *μ*L of the previous ISP-2 broth. Each culture was performed in triplicate. After each stage, strain samples were inoculated on ISP-2 agar and evaluated using the Gram stain protocol to ensure no contamination of the media.

### 2.5. Determination of PVC Biodegradation Capacity

PVC biodegradation was assessed using a compilation of techniques (i.e., weight loss, thermal stability, and by-product analysis) to determine the effect of the selected strains on PVC samples. This compilation aimed to withstand the degradation process using diverse methodologies.

#### 2.5.1. Determination of PVC Weight Loss

PVC weight was measured for every sample before and after 18 days of incubation on MSM, and PVC was the sole carbon source. After incubation, the culture media were centrifuged at 7,000 × *g*for 10 min to remove the biomass. Thereafter, the sample was filtered with a previously weighted Whatman filter paper grade 1 (*ca.* 11 *μ*m). The filter paper was dried at 60°C for 24 h and weighed to determine the remaining PVC on the culture media. The remaining PVC and filtered media were stored for further analyses.

#### 2.5.2. Determination of Changes in the Thermal Stability of Remaining PVC

Thermogravimetric analysis (TGA) was performed to determine the changes in the thermal stability of the remaining PVC after bacterial exposure, as stated by Uscátegui et al. (2019) [28]. TGA was performed using a thermogravimetric analyzer (TGA; Mettler Toledo, Greifensee, Switzerland) at a heat rate of 25°C min^−1^ from 25°C to 600°C in a nitrogen atmosphere with a sample weight of 15 ± 2 mg. The results were compared to those of PVC resin on MSM without bacterial exposure, which served as a control to remove changes in PVC induced by culture conditions.

#### 2.5.3. By-Product Analysis of PVC Degradation by GC-MS and Ionic Exchange Chromatography

The culture media supernatant was analyzed by GC-MS [29] and ionic exchange chromatography [30] to assess any degradation metabolites. For GC-MS, the supernatant was extracted with ethyl acetate at a 1 : 2 ratio, stirred every 15 min for 8 h, and left still for 24 h. The organic phase was separated and injected (1 *μ*L) on a Trace 1300 gas chromatograph (Thermo Scientific, USA) coupled to an ISQ-LT mass spectrometer (Thermo Scientific, USA). The extracted components were separated on a Rxi-5 ms capillary column (60 m × 0.25 mm ID × 0.25 *μ*m film, Restek, USA) based on temperature programming, while the initial temperature of 40°C was kept constant for 5 min, increased to 200°C at a constant rate of 10 °C/min, followed by 260°C at a constant rate of 5°C/min, and finally to 300°C at a constant rate of 10°C/min. The mass analyzer consisted of a single quadrupole, using electron impact ionization at 70 eV. For ionic exchange chromatography, the supernatants were injected into an 883 Basic IC Plus apparatus (Metrohm AG, CH) with a MetroSep A Supp 5–150/4.0 column (Metrohm AG, CH) and analyzed using the anionic analysis method with the MagIC Net 3.3 software (version 3.3, Metrohm AG, CH).

### 2.6. Identification of Selected Strains

The selected strains, which were not previously identified by GIBP [31], were identified using the extraction protocol from GF-1 Bacterial DNA Extraction Kit (Vivantis Technologies, Selangor, Malaysia). The 16S rRNA gene was amplified using polymerase chain reaction (PCR) based on the DNA extracted using oligonucleotides 27F (5′ AGAGTTTGATCMTGGCTCAG 3′) and 1492R (5′ TACGGYTACCTTGTTACGACTT 3′ in a volume of 25 *μ*L as described by Pastrana et al. [27]. Sequences were used to identify closely-related strains in BLAST (Bethesda, USA) and analyzed using the MEGA-X program (Version 10.2.4, USA).

### 2.7. Selection of Culture Conditions with Higher Influence on PVC Biodegradation Capacity

Based on data obtained on standard culture conditions and considering that PVC biodegradation capacity can be influenced by external conditions [32], seven culture conditions were evaluated to determine their effect on PVC biodegradation capacity by strains 208 and 290. This evaluation was performed using a Plackett−Burman design measuring PVC resin weight loss percentage as the response variable.  Table 2 shows the Plackett−Burman design matrix for 12 trials with two levels each. The models obtained were statistically analyzed by a variance analysis (ANOVA) using Minitab 2017 (version 18.1, Pennsylvania, USA). A Pareto diagram was obtained to determine three culture conditions with a larger influence on PVC biodegradation capacity, and the remaining variables were considered as constants on further treatments.

### 2.8. Optimization of Selected Culture Conditions

According to the Pareto diagram, the three culture conditions with higher influence on PVC weight loss were optimized using a Box−Behnken design. The general equation and coefficients were calculated using Minitab 2017 (version 18.1, Pennsylvania, USA). The general equation for the second-order polyonomy is shown in

(1) $$\begin{array}{c} Y_{i}=\beta_{0}+\sum_{\beta i}X_{i}+\sum \beta_{ii}X_{i}^{2}+\sum \beta_{ij}X_{i}X_{j,} \end{array}$$

where *Y*_*i*_ is the estimated response variable and *X*_*i*_*X*_*j*_ are the influential variables on response Y; *β*_0_ is the offset; *β*_*i*_ is the linear coefficient; *β*_*ii*_ is the quadratic coefficient; and *β*_*ij*_ is the interaction coefficient. The optimization model obtained for PVC biodegradation capacity was evaluated at the flask scale, and statistical analysis was performed to determine the suitability of the mathematical relation obtained. The Box−Behnken matrix for 15 trials was obtained with Minitab 2017 (version 18.1, Pennsylvania, USA), and the three selected factors are shown in Table 3.

### 2.9. Statistical Analysis

Data exploration and description were performed using Microsoft Excel 2019 (Microsoft®, USA). Statistical analysis was performed using Minitab 2017 (version 18.1, Minitab, USA). Each experiment was performed in triplicate. Analysis of variance (ANOVA) was used to evaluate the differences between the means, with a level of statistical significance at *p* < 0.05. Post hoc analyses were performed for all data treatments.

## 3. Results

### 3.1. Determination of PVC Biodegradation Capacity

#### 3.1.1. Determination of PVC Weight Loss

**Figure 1:**
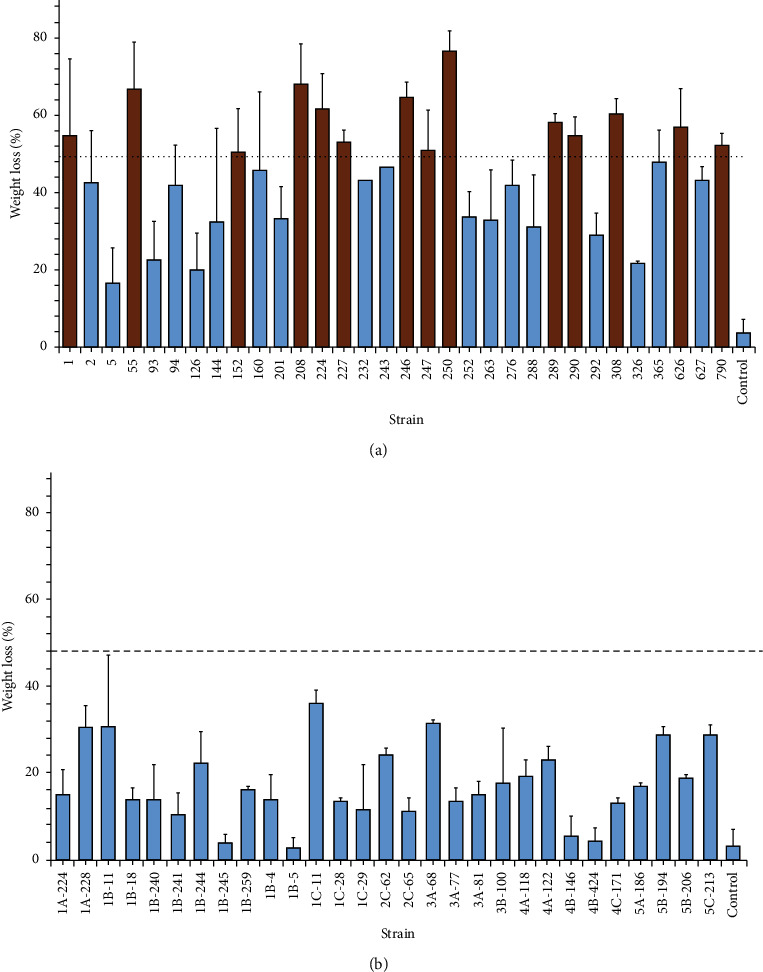
PVC weight loss percentage by each strain on modified ISP-2 broth (a) Arauca isolates and (b) Guaviare isolates. “Control” is the negative control (MSM + PVC with no bacterial exposure). The dotted line represents 50% of the weight loss established limit. Orange bars represent the strains that were further analyzed. All values are presented as mean ± error of data based on experiments performed in triplicate. Bars indicate the standard deviation of data.

Variations in the gravimetric weight of plastic samples can be considered a first approach to determine whether considerable degradation occurs. Thus, the screening stage was performed to determine whether weight loss occurred in the PVC samples after bacterial exposure to selected strains. In this regard, a preliminary screening was conducted for PVC biodegradation by each strain that grew on ISP-2 agar (a total of 61 strains). This screening was conducted as an adaptation phase for bacterial strains and to establish the strains with the potential for PVC biodegradation. Strains with a weight loss higher than 10% were considered positive for PVC biodegradation. Strains that showed a weight loss >50% were further analyzed.  Figure 1 shows that 14 strains could reduce the initial PVC sample weight by more than 50%. These positive strains were isolated from the Arauca riverbank. A culture medium with no inoculum was used as a negative control.

**Figure 2:**
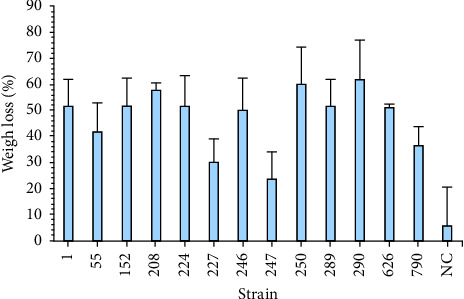
PVC biodegradation as a weight loss percentage by the selected strain on MSM. NC is the negative control (i.e., MSM + PVC resin with no bacterial exposure). The negative control was subjected to the same culture conditions as the experimental treatments. All values are presented as mean ± error of data based on experiments performed in triplicate. Bars indicate the standard deviation of data.

Based on the results of the preliminary biodegradation study on modified ISP-2 broth, the 14 strains with higher PVC biodegradation capacity (>50% weight loss, shown in orange in Figure 1) were tested on MSM with PVC as the sole carbon source (ca. 2 g/L).  Figure 2 shows the PVC biodegradation capacity as a weight loss percentage by each strain after incubation (18 days). The weight loss percentages varied from 25% to 62%. Therefore, some strains (i.e., 250 and 290) were found to biodegrade PVC in both culture media under the same conditions, reaching a maximum of 62% weight loss. The variability of the data shows that a gravimetric method must be performed with sufficient replicates to obtain a lower error in the measurements. In addition, a reassuring method is needed to guarantee the occurrence of degradation [33].

#### 3.1.2. Determination of Changes in the Thermal Stability of Remaining PVC

**Figure 3:**
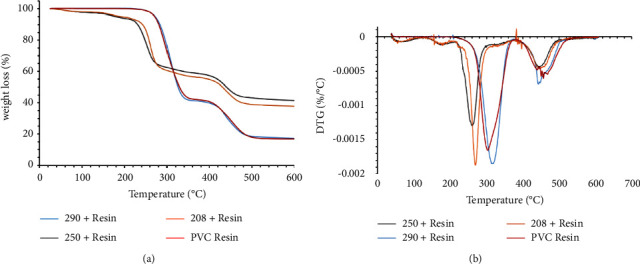
Thermal analysis of remaining PVC (a) TGA curves, and (b) the first order derivative of TGA curves of dried PVC after bacterial exposure with the three selected strains. PVC resin was used as the negative control.

A TGA analysis was performed for PVC samples after bacterial exposure with three strains that showed higher weight loss in previous essays (i.e., 208, 250, and 290) to establish whether the strains had a degradation effect on the PVC backbone. TGA of PVC powder after bacterial exposure revealed lower thermal stability than that of the control, as shown in Figure 3. This thermal stability primarily depends on the polymer chain's length, type, and structure [19].

In Figure 3(b), the first degradation stage, associated with the chloride release found between 250 and 318°C, occurred at lower temperatures when the PVC was incubated with strains 208 and 250 (orange and grey lines) at 280 and 256°C, respectively, than the resin used as the control (312°C). The PVC resin incubated with strain 290 showed higher thermal stability, as the first degradation stage occurred at 318°C.

#### 3.1.3. By-Product Analysis of PVC Degradation by GC-MS and Ionic Exchange Chromatography

**Figure 4:**
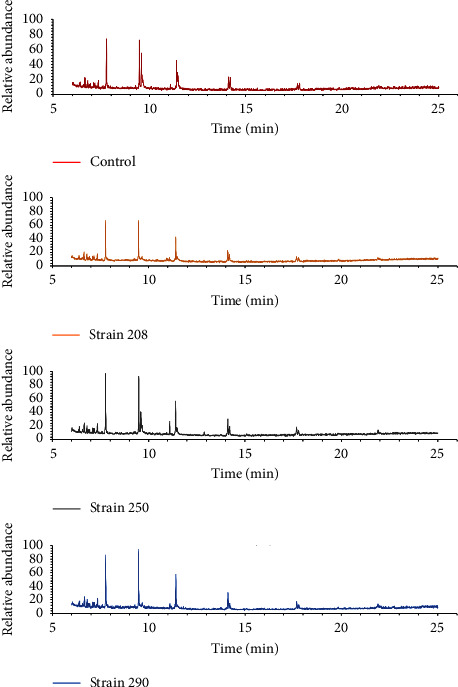
GC-MS chromatogram for the organic extracts of culture media supernatant for each strain. The control is supernatant with no bacterial exposure.

GC-MS analysis revealed similarities between the PVC resin after bacterial exposure and the nontreated resin. However, the main differences were found at 9.6, 11.5, and 14 min, whose relative abundance varied depending on the treatment and strains (Figure 4). These representative peaks were identified to be related to aromatic and alicyclic compounds, such as 1-acetylcur-16-en-20-ol and 9-*t*-butyltricyclo[4.2.1.1(2,5)]decane-9,10-diol, and were found in the supernatant of the culture media from strains 250 and 290.

**Figure 5:**
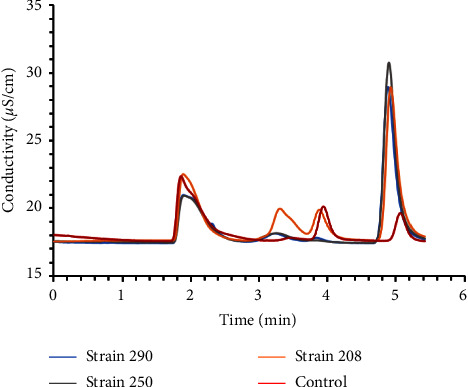
Ion exchange chromatogram for the culture media supernatant containing the selected strains (208, 250, and 290) and culture media with no bacterial exposure as control.

According to the results of ionic exchange chromatography, chloride ions increased in the supernatant of the culture media after bacterial exposure, as shown in Figure 5. Chloride has a retention time of 5 min, where a higher conductivity was observed. Therefore, a higher concentration of chloride was found in samples with bacterial incubation, up to 12 ppm for strain 250 (grey line). A control with no inoculum was used to determine the amount of chlorine in the basal medium, as depicted by the red line. A calibration curve of electrical conductivity (EC) (mS/cm) versus peak area at 5 min was constructed using standard chloride solutions to estimate the concentration of chloride (ppm) by ion exchange chromatography (IEC). It was obtained as a linear equation [Cl^−^] = 0.9824 EC (*R*^2^ = 0.95). Employing the calibration curve, concentrations between 70 and 90% of the chloride amount, expected by stoichiometry, were obtained.

A further analysis was performed using the consortia of the three selected strains. Four combinations were used to ensure that each strain was tested, with a higher degradation expected owing to the consortia [34]. The results (not shown) of weight loss and TGA did not significantly differ from the previous individual evaluation (*p* value = 0.1). Such a finding could indicate that the biodegradation/biotransformation of PVC may occur via the same mechanism in each strain; therefore, the effect is not enhanced. However, competition exists for the substrate, resulting in a nonsignificant change in the desired effect [35].

### 3.2. Identification of Selected Strains

Isolates showing the highest degradation capacity (>50%) were identified by 16S rRNA gene sequencing. The results of the molecular identification of the selected strains are shown in Table 4. Similarly, ≥98.7% was used as the standard for molecular identification of this genus [27].

### 3.3. Selection of Culture Conditions with Higher Influence on PVC Biodegradation Capacity

**Figure 6:**
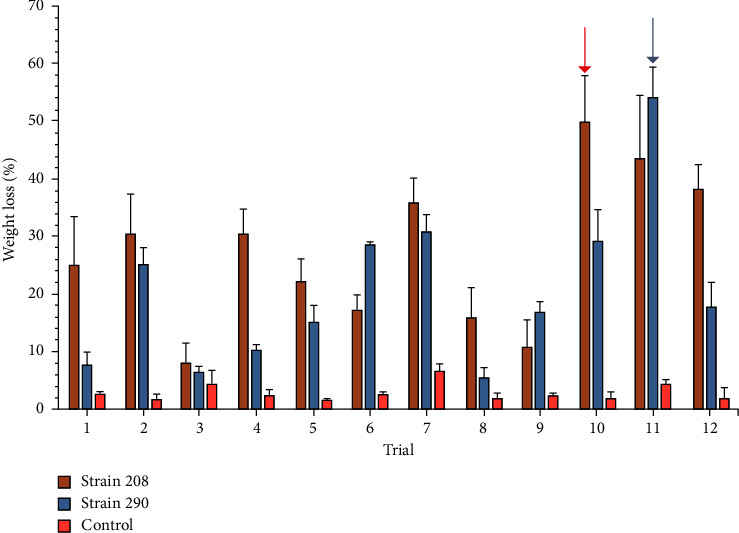
PVC weight loss percentage by each strain on Plackett−Burman design trials. All the values are presented as the mean ± error of data from experiments performed as triplicates. Bars indicate the standard deviation of data. Arrows show the trial with higher PVC weight loss by each selected strain, trial 10 for strain 208 (orange) and trial 11 for strain 290 (blue). Control used was PVC resin on culture media (MSM) with no bacterial exposure.

The results for PVC weight loss for each trial of the Plackett−Burman design are shown in Figure 6. As observed, strains 208 and 290 (orange and blue bars, respectively) showed a variation in the PVC biodegradation, reaching up to 54 ± 5.4% of weight loss. In the case of strain 290, the PVC degradation was achieved at 25°C, 200 rpm, pH of 6, 500 *μ*L of inoculum, concentrations of 0.32 g/L and 2.5 g/L of nitrogen source and PVC, respectively, and a PVC particle size of 150 *μ*m.

Statistical analysis was done using PVC weight loss as the response variable of the Plackett−Burman design. The results of the mathematical relations obtained for each selected strain (i.e., 208 and 290) are shown in Table 5.

**Figure 7:**
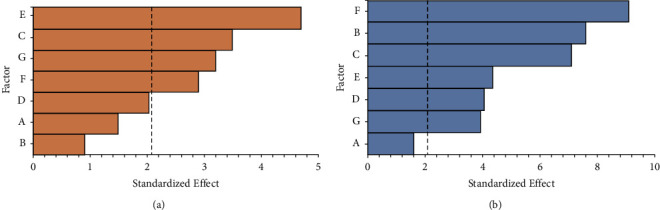
Pareto diagrams of culture conditions influence for selected strains. (a) strain 208 and (b) strain 290. The dotted line represents the significance limit (*α* = 0.05). Temperature (A), agitation rate (B), pH (C), inoculum/volume ratio (D), nitrogen source concentration (E), PVC concentration (F), and PVC particle size (G) are given.

These two mathematical relations showed a suitable model that describes at least 50% of the predicted values of PVC weight loss for the selected strains under controlled culture conditions. The Pareto diagram with the influence of culture conditions on PVC weight loss for each strain is shown in Figure 7.

As shown in Figure 7, for both strains 208 and 290, more than half of the tested culture conditions significantly affect PVC weight loss percentage, with nitrogen source and PVC concentrations the most influential variables for strains 208 and 290, respectively. Each significant factor showed a *p* value lower than 0.01. For further explanation, the *p* values obtained in the ANOVA analysis for each tested culture condition are shown in Table S1.

### 3.4. Optimization of Selected Culture Conditions

**Figure 8:**
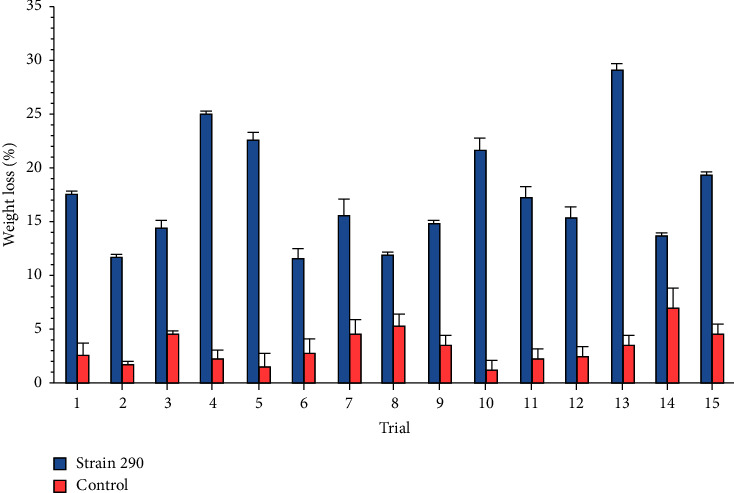
PVC weight loss percentage by each strain on Box−Behnken design trials. All the values are presented as the mean ± error of data from experiments performed as triplicates. Bars indicate the standard deviation of data. Control used was PVC resin on culture media with no bacterial exposure under the same fermentation time.

Considering the suitability of the mathematical relations obtained, strain 290 was selected to optimize the three culture conditions with a higher effect on PVC weight loss percentage (i.e., PVC concentration, agitation rate, and pH). The results for PVC weight loss for each trial of the Box−Behnken design are shown in Figure 8. With this design, a PVC weight loss percentage up to 24% ± 0.5% was achieved, considering the modification on each culture condition that significantly affected the response variable.

### 3.5. Mathematical Relation of Culture Conditions' Optimization and PVC Weight Loss

After the Box−Behnken optimization, the general equation obtained for the optimization model is shown in

(2) $$\begin{array}{c} Y\left(\%\right)=234.1-32.8F-2.338B+12.5C+10.27F^{2}+0.00685B^{2}-2.175C^{2}-0.2108FB+0.81FC+0.0716BC, \end{array}$$

where *Y* is the estimated response variable, *F* is the PVC concentration, *B* is the agitation rate, and *C* is the pH. For this mathematical relation, the determination coefficients (*R*^2^) are shown in Table 6.

**Figure 9:**
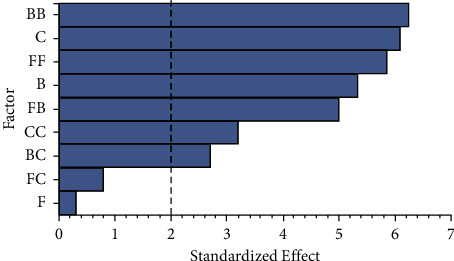
Pareto diagram for Box−Behnken optimization of culture conditions with strain 290. The dotted line represents the significance limit (*α* = 0.05). PVC concentration (F), agitation rate (B), and pH (C). Combined letters represent the interaction between culture conditions.

Additionally, the Pareto diagram for each coefficient of the Box−Behnken optimization is shown in Figure 9. For strain 290, the PVC concentration and the interaction between PVC concentration and pH did not significantly affect the PVC weight loss percentage. The remaining variables showed a *p* value lower than 0.01 and were held constant throughout this stage with the values for standard conditions.

After Box−Behnken optimization, the values obtained for each culture condition that allows a maximum PVC weight loss were then calculated (Table 7). Hence, the optimum PVC concentration was observed to be the highest one (3.75 g/L), while the pH and agitation rate should be close to the lowest values (pH = 6.06 and 150 rpm). These optimized culture conditions led to a PVC biodegradation of 30.15% (predicted response). Such culture conditions were subsequently replicated at the flask scale in triplicate, whose obtained results are shown in Table 8. Such conditions were successfully replicated. In this regard, the experimental and predicted response were 29.55 and 31.16%, involving a reasonable estimated error (i.e., 5.12%) and a suitable determination coefficient (*R*^2^ = 0.98).

## 4. Discussion

PVC is one of the most polluting plastic wastes worldwide. To date, no strategies have been raised to be employed as an ideal option to address this problem. Biotransformation/biodegradation has been identified as an approach that can contribute significantly to solving this challenge [12]. In this study, we identified 14 freshwater-derived *Streptomyces* strains that showed a high yield (> 50% weight loss) for PVC biodegradation. In gravimetric measurements (a standard approach that is widely used [13]), these strains had weight loss percentages between 17% and 75% on a glucose-supplemented culture medium (e.g., ISP-2) and between 24 and 62% on a culture medium with PVC as the sole carbon source. This result is considerably higher than that of other studies on PVC biodegradation. Giacomucci et al. established a biodegradation rate of 19% using *Pseudomonas citronellolis* and *Bacillus flexus* [19]; this can be attributed to the PVC type used for each test, as the researchers used PVC films. Bacteria tend to biodegrade plasticizers and not polymers primarily [34]. Additionally, powdered samples can achieve higher biodegradation [13, 14]. The powdered samples used in this study were PVC resin without plasticizers.

Other studies revealed similar results using *Streptomyces* for PET biodegradation (68% weight loss on MSM) [13]. According to Farzi et al., smaller particle sizes can produce a higher biodegradation rate [13]; this can explain the high PVC biodegradation capacity, measured as weight loss, using a particle size up to 150 *μ*m, compared to PET (particle size up to 220 *μ*m), even when PVC has a higher complexity to degrade [3]. Additionally, strains grown in ISP-2 broth had notably higher biofilm production than those grown in the PVC-supplemented broth; this could cause a higher PVC weight loss as the bacterial strains grew better on this culture media [23]. As MSM is a basal culture medium, the growth conditions can be limited, and biodegradation can occur at a slower rate [35].

Weight loss has been used in many studies as a benchmark for identifying the degradation of a material [13]. However, this approach has several challenges owing to its inability to discriminate false positives or inconclusive outcomes, such as an increase in the sample weight [36]. The degradation of a material involves the reduction of molecular weight, release of by-products, and modification of mechanical or physical properties [26]. We incorporated weight loss, by-product analysis of the supernatant, and thermogravimetric measurements as strategies to obtain orthogonal evidence of PVC degradation/biotransformation. Weight loss can provide information on the possible chain shortening caused by *Streptomyces* to PVC. However, by-product analysis and TGA support the possibility that chain modification or shortening occurs in PVC, as biodegradation has been reported to involve the reduction of the main backbone to its constituent monomers [5].

Based on the TGA results, an expected behavior was obtained for strains 208 and 250, as the bacterial exposure influenced the thermal stability of the nontreated PVC resin, lowering the temperature at which the degradation stages occur [34]. The decrease in stability can be due to a chain length reduction or a polymer structure modification due to biodegradation [19, 37]. For strain 290, there was a change in the degradation stages of the PVC resin; however, the temperatures were higher than those obtained from the nontreated PVC. As the resin had no additives, the behavior of strain 290 was not as expected [34]. This finding might be due to the addition of metabolites produced in the culture medium by this strain, which allowed a higher temperature before the degradation stages [34, 38]. This result also explained the yellowish color (Figure S1), which indicated that the resin was exposed to strain 290. Also, this may indicate that the degradation mechanism by strain 290 may differ from the other tested strains [39].

Evidently, for strains 208 and 250, weight loss occurred at approximately 100°C, which could be associated with water retention by the polymeric matrix [3]; this is despite the retention of samples in a dry state until analysis. Ultimately, evaporation occurred at approximately 100°C. Therefore, a change in the polarity of the PVC resin occurred, as this resin tends to be hydrophobic when its backbone is not manipulated [3, 40]. If the PVC backbone chain is “broken” by microorganisms, the chain will no longer be saturated, and alkane structures will occur, leading to a hydrophilic behavior of the material [41]. In addition, the first stage of degradation is associated with chloride release (usually hydrochloride), and the second stage is related to modifying the carbon-carbon bonds [8]. In this regard, the resin used as control had almost 58% weight loss due to the chloride release. By contrast, strains 208 and 250 had a weight loss of 38% in the same degradation stage. The reduction in weight loss due to chloride release on strains 208 and 250 suggests that there is less amount of chloride on the polymeric matrix compared to the control [42].

GC-MS analysis revealed the presence of an aromatic compound, i.e., 1-acetylcur-16-en-20-ol, and a tricyclic aliphatic compound, i.e., 9-t-butyltricyclo[4.2.1.1(2,5)]decane-9,10-diol, as by-products in the culture media supernatant after bacterial exposure. Although the aromatic or alicyclic compounds were not directly related to the PVC transformation, they were found to be by-products of polyethylene degradation [43]. As the polymers' complexity is high, these compounds' presence may indicate that the by-products, after a plausible dechlorination phase, are oxidized to carboxylates, which are further condensed and/or cyclized by Claisen reactions, and even subsequently aromatized [44]. However, these would also be secondary products generated by the strain metabolism (constitutive or induced) using the available carbon sources [44]. In this sense, aromatic and alicyclic compounds are related to the degradation of a wide variety of synthetic plastics based on aliphatic chains [3]. Hence, the presence of these compounds may indicate that degradation of the polymer backbone occurs after bacterial exposure and leads to aromatic or alicyclic moieties.

Ionic exchange chromatography was performed to understand the possible PVC degradation pathways. Hence, dechlorination was observed after the TGA results, despite a low release of chloride in the culture media was expected. The data in Figure 5 partially support the hypothesis that chloride ions are released within the culture media. Thus, chloride was increased in the culture media supernatant of the test strains. In this analysis, strain 250 showed a higher chloride concentration in the culture media (i.e., up to 12 ppm), and strains 208 and 290 had almost the same concentration of chloride released (i.e., up to 10 ppm). Additionally, if degradation occurs during the first stage of dechlorination of the PVC chain, the release of chloride into the culture medium is expected [30, 45]. The chloride release could indicate the biological pathway for the biodegradation of PVC by *Streptomyces* strains. In this regard, dehalogenases are enzymes with great potential in the bioremediation of organochlorides (e.g., vinyl chloride monomer) [46]. In fact, the occurrence of dehalogenase enzymes has been reported in *Streptomyces* [47]. For example, works such as those of Ito et al. [48] and Alvarez et al. [49] reporting organochlorine-degrading *Streptomyces* strains (i.e., DDT and lindane, respectively) illustrate the potential of *Streptomyces* in the degradation of halogenated organics. At this point, it is important to note that further studies should involve analyzing the implications of chloride release from these degradations. However, under this context, identifying *Streptomyces* as biodegraders opens up engaging scenarios. For instance, *Streptomyces* also encodes halogenases [50] (e.g., haloperoxidases and flavin-dependent halogenases), which can exploit the available chloride for the bioproduction of value-added metabolites such as antibiotics [51] and pesticides [52], which enhances the sustainable exploitation of this bioresource.

From the 14 strains that showed a PVC weight loss higher than 50%, none of the closely related neighbors have been reported as potential PVC degraders, either on film or powdered samples. Based on the phylogenetic analysis, almost all identified strains were in the same clade. Three strains were not identified at the species level and were instead identified as genus (*Streptomyces* sp.), which could be due to the nature of *Streptomyces.* It is difficult to thoroughly identify *Streptomyces* using gene 16S [27] as this gene can be closely related between *Streptomyces* species. Further studies, such as complete genome studies, need to be performed to identify these strains at the species level. To the best of our knowledge, these identified species have not been reported as synthetic polymer degraders. However, *S. albospinus* (the closest neighbor for strain 290) and *S. kasugaensis* (the closest neighbor for strain 208) have been reported as natural polymer degraders (i.e., cellulose) [53] and biocontrol agents [54], respectively. Such a finding highlights the potential of these strains to biodegrade synthetic plastics.

The effect of culture conditions on PVC biodegradation capacity for two *Streptomyces* strains was evaluated, as culture conditions mainly influence bacterial behavior upon determined substrates [55]. Some researchers have determined that culture conditions such as carbon and nitrogen source concentration, pH, and temperature influence bacterial behavior (i.e., enzyme production) [55] and must be considered in biological processes. Moreover, conditions like plastic particle size influence plastic biodegradation capacity [13]. Hence, the influence of seven culture conditions (i.e., temperature, agitation rate, pH, inoculum/volume ratio, nitrogen source concentration, PVC concentration, and PVC particle size) on PVC weight loss percentage was determined using a Plackett−Burman design. These culture conditions were selected based on previous research performed by the GIBP research group [23, 27]. Plackett−Burman design allows more easily identifying the most positive influential factors on a determined response variable [56, 57]. It also allows comprehending and mathematically relating the effect of factors on the desired variable [56].

Although the Plackett−Burman design helps know the effect of individual factors, the interactions between those factors are not considered [58], which may affect the reduction of the PVC biodegradation capacity, as the interactions between factors may have a higher impact on the response variable than the individual factors. Also, it is notable that some of the factors on the Plackett−Burman, such as pH for strains 290, have a significant effect, but it turned out to be a negative one. These effects and relations need to be considered as part of the optimization. As shown in Figure 7, for strain 208, the main culture conditions with a significant positive effect on PVC weight loss percentage were, from higher to lower significant effect, nitrogen source concentration, pH, PVC particle size, and PVC concentration. For strain 290, these significant culture conditions were determined as PVC concentration, agitation rate, pH, nitrogen source concentration, inoculum/volume ratio, and PVC particle size. The first three culture conditions listed for these strains had a significant effect at above 99.9% of PVC weight loss percentage.

As these strains were isolated from Arauca riverbanks [31], it is expected that some environmental conditions need to be replicated at the flask scale to obtain better or more relevant results [59]. According to this, it is anticipated that for both strains (208 and 290), pH is one of the most influential variables on the PVC weight loss, as lower pH values tend to increase the strains activity, as shown in the optimization stage, were pH values of 6.06 showed a suitable activity on the response variable. The variables with confidence levels greater than 95% were considered as influencing PVC weight loss percentage. The variables that did not meet the confidence levels were considered as insignificant to PVC weight loss percentage and remained constant for the optimization with the Box−Behnken design at a standard level [27]. Box−Behnken design was selected due to the reduction of trials on the optimization stage, as the region where the experiments are carried out does not consider extreme conditions [60]. This allows for saving time and resources with each trial. The optimal conditions found with the Box−Behnken design showed reliability in predicting future values of PVC weight loss percentage by strain 290 under controlled conditions. The PVC weight loss percentage was determined at 29.55% on a practical level, while the theoretically predicted value was 30.16%, as shown in Table 8. The excellent correlation between predicted and measured values validates the model and the existence of a local maximum.

Even when the optimization showed a reasonable prediction, the PVC weight loss percentage tended to decrease against standard conditions. This outcome can be due to demanding conditions using PVC as the sole carbon source [13], the narrow range of selected culture conditions, or finding a local maximum on the degradation process [57]. However, in this case, the second-order model shows a correlation coefficient of 0.83, which is under an acceptable range for biological materials [61]. Additionally, an analysis performed with the desirability function showed that this value is 0.95. As the value is higher than 0.7, it indicates that a suitable optimization was performed considering the experimental data obtained [62]. Moreover, this desirability function lets us know if the maximum value obtained is maximum. As this value is between 0 and 1 (0.95), this result means that the value obtained is between the minimum and maximum values [61], hinting that a local maximum could be obtained. Hence, a lower PVC biodegradation capacity can be showed in this optimization stage.

Other approaches such as kinetic modeling have shown suitable fits with the expected weight loss percentage with other plastics, such as PET [13], where the culture condition changed was the plastic sample particle size. The mathematical relation obtained in this research from the optimization shows that black-box models can also allow a suitable fit for biodegradation processes. So far, and to our knowledge, no mathematical relation between culture conditions and PVC weight loss percentage by *Streptomyces* strains has been reported. This study could be the first approach to a scale-up process for PVC biodegradation.

Further studies are recommended to better understand the pathway, or the enzymes released in the biodegradation process, which will help other efforts to target the production of these enzymes and discover new species with PVC biodegradation capability. Likewise, the whole-genome sequence can be performed to identify whether the species share genes that could be related to PVC biodegradation. From this perspective, identifying these *Streptomyces* strains is a worthwhile opportunity to find sustainable options for the bioremediation of plastic pollution. Even more so nowadays, the advances in areas such as molecular biology and genetic engineering have made it possible from the application of biotechnological tools to make improvements in the catalytic capacity of these enzymes and thus convert these approaches into viable application alternatives. One such example is the work of Austin et al. [63], who succeeded in improving the catalytic activity of a bacterially derived PETase. Such a study was only possible due to the discovery of *Ideonella sakaiensis* 201-F6 by Yoshida et al. [64].

## 5. Conclusions

The potential of *Streptomyces* strains to induce PVC biodegradation was evaluated in this study. Sixty-one freshwater-derived *Streptomyces* strains were tested for their potential to induce biodegradation of powdered PVC resin under aerobic conditions. Novel high-yield PVC biodegradation was achieved using freshwater-derived *Streptomyces.* In the preliminary screening, 14 strains showed PVC biodegradation capacity higher than 50%, measured as weight loss after incubation. Among these strains, three strains showed the highest biodegradation capacity on MSM with PVC as the sole carbon source. Using the 16S rRNA gene, the three strains were identified as *Streptomyces* with closest neighbors, such as *S. albospinus* (290), *S. chattanoogensis* (250), and *S. kasugaensis* (208). By-product analysis and TGA revealed that a decrease in the polymer chain occurred and affected the stability of the PVC. Our findings highlight three *Streptomyces* strains as novel PVC biodegradation species and their potential use as a complement to the PVC biodegradation method. A mathematical relation between culture conditions and PVC weight loss by strain 290 was found, showing an accuracy of 95% and a correlation coefficient of 98%. To the best of our knowledge, this is the first study on PVC biodegradation by *Streptomyces* strains.

## Figures and Tables

**Table 1:** Culture media components.

Culture media	Component	Concentration (g/L)
ISP-2 agar	Yeast extract	2
	Malt extract	5
	Glucose	2
	Agar	20

Modified ISP-2 broth	Yeast extract	2
	Malt extract	5
	Glucose	0.5
	PVC resin	1.5^1^
	Tween 80	0.1%

Mineral salt medium (MSM)	(NH_4_)_2_SO_4_	0.741
	NaNO_3_	0.21
	KH_2_PO_4_	0.9
	Na_2_HPO_4_	1.8
	Ca(NO_3_)_2_	0.06
	MgSO_4_ 7H_2_O	0.15
	PVC resin	2^1^

^1^PVC resin on culture media is related to the resin amount added separately to each culture due to the low solubility of PVC in the culture medium.

**Table 2:** Plackett−Burman design matrix for PVC biodegradation capacity with each selected strain.

Trial		1	2	3	4	5	6	7	8	9	10	11	12	Level	+1	−1
Factors^+^	A	+1	+1	−1	+1	+1	+1	−1	−1	−1	+1	−1	−1	°C	35	25
	B	−1	+1	+1	−1	+1	+1	+1	−1	−1	−1	+1	−1	rpm	200	150
	C	+1	−1	+1	+1	−1	+1	+1	+1	−1	−1	−1	−1	pH	8	6
	D	−1	+1	−1	+1	+1	−1	+1	+1	+1	−1	−1	−1	v/v	1/2	1/4
	E	−1	−1	+1	−1	+1	+1	−1	+1	+1	+1	−1	−1	g/L	0.74	0.32
	F	−1	−1	−1	+1	−1	+1	+1	−1	+1	+1	+1	−1	g/L	3.75	2.50
	G	+1	−1	−1	−1	+1	−1	+1	+1	−1	+1	+1	−1	*μ*m	150	75

^+^Temperature (A), agitation rate (B), pH (C), inoculum/volume ratio (D), nitrogen source concentration (E), PVC concentration (F), and PVC particle size (G) are given.

**Table 3:** Box−Behnken matrix for culture conditions' optimization for PVC biodegradation capacity.

Trial		1	2	3	4	5	6	7	8	9	10	11	12	13	14	15
Factors^**+**^	A	0	0	−1	0	1	1	0	0	−1	0	0	1	1	0	−1
	B	1	0	0	−1	1	0	1	−1	1	0	0	0	−1	0	−1
	C	−1	0	−1	−1	0	1	1	1	0	0	0	−1	0	0	0

^+^Three most influential factors that affect PVC weight loss were taken from the Pareto diagram. A, B, and C represent the three most influential culture conditions depending on the tested strain. 1, 0, and −1 represent the levels of each culture condition, being (−1) the lowest level, (0) the middle level, and (1) the highest level.

**Table 4:** Molecular identification of strains with PVC weight loss higher than 50%.

Strain	Closest neighbors	Identity (%)
1	Streptomyces graminearus	99.9
55	*Streptomyces lunalinharesii*	99.1
152	*Streptomyces albospinus* ^*+*^	98.7
208	*Streptomyces kasugaensis* ^*+*^	98.7
224	*Streptomyces chattanoogensis*	99.1
227	*Streptomyces* sp.^+^	98.7
246	*Streptomyces graminisoli* ^*+*^	98.7
247	*Streptomyces* sp.^+^	98.7
250	*Streptomyces chattanoogensis*	98.9
289	*Streptomyces* sp.^*+*^	98.7
290	*Streptomyces albospinus* ^*+*^	98.7
308	*Streptomyces lydicus* ^*+*^	98.7
626	*Streptomyces lunalinharesii* ^*+*^	98.7
790	*Streptomyces iranensis* ^*+*^	98.7

^+^Strains were previously identified in GIBP studies [31].

**Table 5:** Mathematical relation of culture conditions and PVC biodegradation capacity for each selected strain.

Strain	Regression equation^+^	*R* ^2^	*R* ^2^ (adjust)	*R* ^2^ (predict)
208	Y(%) = 51.4 + 0.389A–0.044B − 5.24 C − 23.8D − 31.55E + 6.4F + 0.1261G	0.6747	0.5927	0.5613
290	Y(%) = −2.2–0.273A + 0.2454B − 5.778C − 26.7D − 17.19E + 12.17F + 0.0842G	0.8902	0.8627	0.8184

^*∗*^Weight loss percentage (Y), temperature (A), agitation rate (B), pH (C), inoculum/volume ratio (D), nitrogen source concentration (E), PVC concentration (F), and PVC particle size (G) are given.

**Table 6:** Determination coefficients for Box−Behnken optimization model.

*R* ^2^	*R* ^2^ (adjust)	*R* ^2^ (predict)
0.8399	0.7987	0.7297

**Table 7:** Optimized culture conditions for a maximum PVC weight loss.

Factor	F (g/L)	B (rpm)	C	Y (%)
Optimized value	3.75	150	6.06	30.155

PVC concentration (*F*), agitation rate (*B*), pH (*C*), and predicted response (*Y*) are given.

**Table 8:** Experimental response for culture conditions' optimization.

Experimental response (%)	Standard deviation	Theoretical response (%)	*R* ^2^ (estimated)	Error (%) (estimated)
29.55	0.04	31.16	0.98	5.12

## Data Availability

The data used to support the findings of this study are provided within this article. However, any required further information can be provided by the corresponding authors upon request.

## Supplementary Materials

Supplementary Materials Figure S1: yellowish color on culture medium of strain 290 on MSM. Table S1: ANOVA table for each of the tested culture conditions on Plackett−Burman design. A significance level of 0.05 was used.
