# Supplementary material for: Freshwater-Derived Streptomyces: Prospective Polyvinyl Chloride (PVC) Biodegraders
Source: ScientificWorldJournal. 2022 Nov 14;2022:6420003. doi: 10.1155/2022/6420003 (PMC9678452; doi:10.1155/2022/6420003)
Supplement: Supplementary Materials — Figure S1: yellowish color on culture medium of strain 290 on MSM. Table S1: ANOVA table for each of the tested culture conditions on Plackett−Burman design. A significance level of 0.05 was used. [file 6420003.f1.docx]

Freshwater-derived *Streptomyces*: prospective polyvinyl chloride (PVC) biodegraders

**Maria Fernanda Rodríguez-Fonseca^1,3^, Sonia Ruiz-Balaguera^2^, Manuel Fernando Valero^4^, Jeysson Sánchez-Suárez^3^, Ericsson Coy-Barrera^5^ and Luis Eduardo Díaz^3,^***

The supplementary material for the paper is presented below.


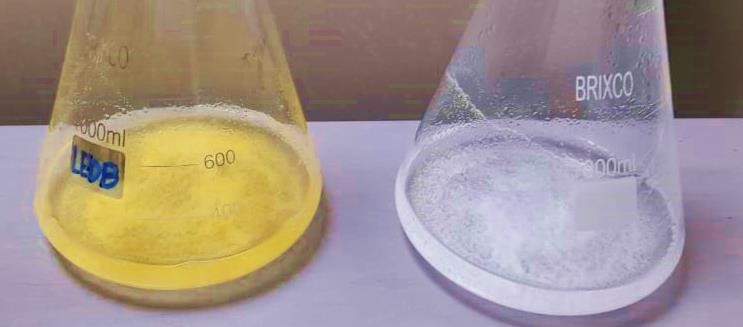


(a) (b)

**Figure S1**. Yellowish color on culture medium of strain 290 on MSM. (a) represents the culture medium incubated with PVC and strain 290, and (b) is the negative control used, MSM with PVC and no bacterial inoculation.

**Table S1.** ANOVA table for each of the tested culture conditions on Plackett-Burman Design. A significance level of 0.05 was used.

| **Source** |  | **Strain 208** | |  | **Strain 290** | |
| --- | --- | --- | --- | --- | --- | --- |
|  |  | **F-Value** | **P-Value** |  | **F-Value** | **P-Value** |
| **Model** |  | 8,28 | 0,001 |  | 2,42 | 0,002 |
| **Linear** |  | 8,28 | 0,002 |  | 2,42 | 0,016 |
| **Temp.** |  | 1,77 | 0,194 |  | 2,56 | 0,121 |
| **rpm** |  | 0,57 | 0,457 |  | 1,82 | 0,001 |
| **pH** |  | 2,87 | 0,001 |  | 5,96 | 0,005 |
| **v/v** |  | 4,15 | 0,051 |  | 5,33 | 0,001 |
| **g/L [N]** |  | 0,58 | 0,011 |  | 7,93 | 0,002 |
| **g/L [C]** |  | 7,51 | 0,011 |  | 9,63 | 0,001 |
| **µm** |  | 0,49 | 0,003 |  | 3,71 | 0,001 |
| **Error**  **Lack-of-**  **Fit**  **Pure**  **Error**  **Total** |  | 8,3 | ≤0.001 |  | 11,77 | ≤0.001 |
